# Integrative multi‐omics epigenome‐wide association study identifies dysregulated DNA methylation near oligodendrocyte genes that associate with tau levels in the AD brain

**DOI:** 10.1002/alz.087790

**Published:** 2025-01-03

**Authors:** Stephanie R Oatman, Joseph S. Reddy, Xue Wang, Zachary Quicksall, Amin Atashgaran, Floor Vanelderen, Jeremiah Bergman, Ozkan Is, Minerva M. Carrasquillo, Chia‐Chen Liu, Yu Yamazaki, Thuy Nguyen, Michael G. Heckman, Na Zhao, Michael DeTure, Melissa E. Murray, Guojun Bu, Takahisa Kanekiyo, Dennis W. Dickson, Mariet Allen, Nilüfer Ertekin‐Taner

**Affiliations:** ^1^ Mayo Clinic, Jacksonville, FL USA; ^2^ Department of Neuroscience, Mayo Clinic, Jacksonville, FL USA

## Abstract

**Background:**

Alzheimer’s disease (AD) is neuropathologically characterized by amyloid‐β (Aβ) plaques and tau neurofibrillary tangles often quantified by Thal phase and Braak stage, respectively. Aβ also frequently deposits in the cerebrovasculature with severity categorized by a cerebral amyloid angiopathy (CAA) score. These and related measures often show high variability within AD suggesting distinct underlying mechanisms. We hypothesize that, within the AD brain, neuropathology and levels of core AD‐related proteins are influenced by variations in DNA methylation (DNAm). To test this, we performed epigenome‐wide association studies (EWAS) using DNAm measures from the temporal cortex (TCX) and cerebellum (CER) with AD‐related neuropathologic measures (Braak, Thal, CAA) and brain biochemical levels of five proteins (apoE, Aβ40, Aβ42, tau, p‐tau).

**Methods:**

DNAm from neuropathologically‐confirmed AD cases was measured by reduced representation bisulfite sequencing (RRBS) from 471 TCX samples, 200 of which also had CER RRBS. TCX levels of five AD‐related proteins from three tissue fractions (buffer‐, detergent‐, and in‐soluble) were measured previously by ELISA (Liu 2020). CpG methylation (CpGm) was binned by a 15‐state chromatin model (Kundaje 2015) and averaged into CpGm clusters (rCpGm). rCpGms were tested for association with each AD endophenotype and expression levels of nearby genes through multi‐variable linear regression. Replication of significant rCpGms was performed in two independent datasets with AD‐related endophenotypes (Shireby 2022, De Jager 2014).

**Results:**

Our innovative binning method demonstrated biologically relevant CpGm patterns. We found epigenome‐wide significant associations primarily with tau‐related endophenotypes including 93 that had significant and concordant associations in the replication datasets. These rCpGms also significantly associated with expression of nearby genes showing enrichment in oligodendrocyte marker genes including those related to myelination. *In vitro* validation of tau effects on oligodendrocyte gene expression through DNAm is ongoing.

**Conclusions:**

Although all endophenotypes tested are core to AD pathophysiology, our results suggest each has a distinct epigenetic architecture underlying their variability in the AD brain. In particular, we found evidence of DNAm variability associating with brain oligodendrocyte gene expression and TCX tau levels. By discovering AD brain endophenotype‐specific DNAm changes, we can identify core components of complex mechanisms revealing important biological insights into AD pathophysiology.

